# Statins—From Fungi to Pharmacy

**DOI:** 10.3390/ijms25010466

**Published:** 2023-12-29

**Authors:** Anna Sadowska, Patryk Osiński, Alicja Roztocka, Karolina Kaczmarz-Chojnacka, Ewa Zapora, Diana Sawicka, Halina Car

**Affiliations:** 1 Department of Experimental Pharmacology, Medical University of Bialystok, Szpitalna 37, 15-295 Bialystok, Poland; diana.sawicka@umb.edu.pl (D.S.); halina.car@umb.edu.pl (H.C.); 2 Student’s Pharmacological Club, Lazarski University, Świeradowska 43, 02-662 Warsaw, Poland; 44452@lazarski.pl (P.O.); 44463@lazarski.pl (A.R.); 44409@lazarski.pl (K.K.-C.); 3 Department of Silviculture and Forest Use, Institute of Forest Sciences, Bialystok University of Technology, Wiejska 45E, 15351 Bialystok, Poland; e.zapora@pb.edu.pl

**Keywords:** statins, fungi, cholesterol, HMG-CoA reductase

## Abstract

Statins have been used in the treatment of hyperlipidemia, both as monotherapy and in combination therapy. Natural fermentation processes of fungi such as *Monascus* spp., *Penicillium* spp., *Aspergillus terreus*, and *Pleurotus ostreatus* have given rise to natural statins. Compactin (mevastatin), the original naturally occurring statin, is the primary biotransformation substrate in the manufacturing process of marketed drugs. Statins are classified into natural, semi-synthetic derivatives of natural statins, and synthetic ones. Synthetic statins differ from natural statins in their structural composition, with the only common feature being the HMG-CoA-like moiety responsible for suppressing HMG-CoA reductase. Statins do not differ significantly regarding their pleiotropic and adverse effects, but their characteristics depend on their pharmacokinetic parameters and chemical properties. This paper focuses on describing the processes of obtaining natural statins, detailing the pharmacokinetics of available statins, divided into natural and synthetic, and indicating their pleiotropic effects.

## 1. Introduction

Many natural products have been the source of interest in drug design, including several dozen mushroom species, especially in Asian countries, where they serve to obtain medicinal preparations as well as dietary supplements. Examples of drugs of fungal origin include statins, which for many years were the sole source of statins.

Before the discovery of statins, the choice of cholesterol-lowering drugs was restricted. Nicotinic acid and fibrates were used to lower cholesterol and triglycerides. However, these medications have only mildly lowered cholesterol levels. In contrast, the use of cholestyramine has been highly effective in treating patients with high cholesterol concentrations, although many patients have tolerated it unfavorably [1].

Statins represent a class of cholesterol-lowering agents used for the treatment of dyslipidemia and to reduce atherosclerotic cardiovascular disease (ASCVD) risk. They are considered the first-choice drug since they decrease morbidity and mortality in patients with an increased risk of ASCVD. Their broad effects on the lipid profile, together with their cardioprotective efficacy, make statins one of the most frequently prescribed medicines worldwide. The statins are divided into natural, their derivatives, and synthetically produced [2].

The aim of this review is to describe the obtaining of natural fungal statins and provide detailed information on the pharmacokinetics and pleiotropic properties of natural and synthetic statins, as well as side effects and drug interactions.

## 2. The Discovery of Statins

The statins were discovered by Akira Endo and his co-workers at the Sankyo pharmaceutical company in Japan. Endo was inspired by Alexander Fleming’s discovery of penicillin, and he focused his research career on fungal enzymes and their potential use in medicine. After the most important enzyme for cholesterol synthesis, HMG-CoA reductase (HMGR), was discovered in 1966, Endo sought HMG-CoA reductase inhibitors, which would become the natural targets. He surmised that the blocking of this enzyme was a protective mechanism used by the molds to compete with other microorganisms, which required sterol and other isoprenoids derived from mevalonate to grow [3]. After screening more than 6000 fungi for their ability to inhibit cholesterol synthesis using a method based on rat liver membranes that convert radioactive acetate to cholesterol, it was revealed that a blue-green mold isolated from rice and named Penicillium citrinum showed significant inhibitory effects on HMG-CoA reductase [4]. In July 1973, three active metabolites were obtained from this mold, and the most potent was ML-236B, which was later called compactin or mevastatin. It turned out that compactin and mevalonate demonstrated similar structures [3]. 

The first in vivo experiments carried out on rats were not effective because of the low levels of LDL lipoprotein in these animals. Endo and his colleagues [3] then went on to test compactin on dogs, hens, rabbits, and monkeys, and the efficacy of their compound was confirmed by a reduction in blood cholesterol levels. In February 1978, Akira Yamamoto, a doctor at Osaka University Hospital, carried out a treatment with compactin on an 18-year-old woman with severe hypercholesterolemia. After using compactin at a dose of 500 mg per day for two weeks, the patient’s cholesterol level decreased from 1000 mg/dL to about 700 mg/dL [3,5]. Unfortunately, after two weeks of treatment, the patient was diagnosed with muscular dystrophy, and the levels of transaminases in her blood increased. Discontinuation of compactin treatment halted the undesirable effects [3,6]. A Japanese company began clinical trials of compactin in November 1978, and all the hospitals that took part in them gave compactin a positive rating [3]. In the same year, Alfred W. Alberts led a team of scientists who independently discovered and examined a potent inhibitor of HMGR [7]. A compound was isolated from the fungus *Aspergillus terreus* and named mevinolin [8]. One year later, Endo obtained monacolin K, another inhibitor of cholesterol synthesis derived from the fungus Monascus ruber [9]. Mevinolin and monacolin K (marketed as lovastatin) were found to be the same compound. These substances had similar biological properties to compactin but differed in chemical structure, possessing an additional methyl group. In 1980, a clinical trial was initiated with lovastatin. However, the trial was soon discontinued due to the suspension of compactin development. This was caused by lymphomas detected in dogs that received doses of 100 or 200 mg/kg/day for two years. Compactin was quite active in patients with severe hypercholesterolemia at low doses of just 1 mg/kg/day, about 200 times lower than in the dog study. Consequently, these studies allowed for the estimation of effective and safe doses of compactin, which enabled this research to resume afterward [3,10,11].

**Figure 1:**
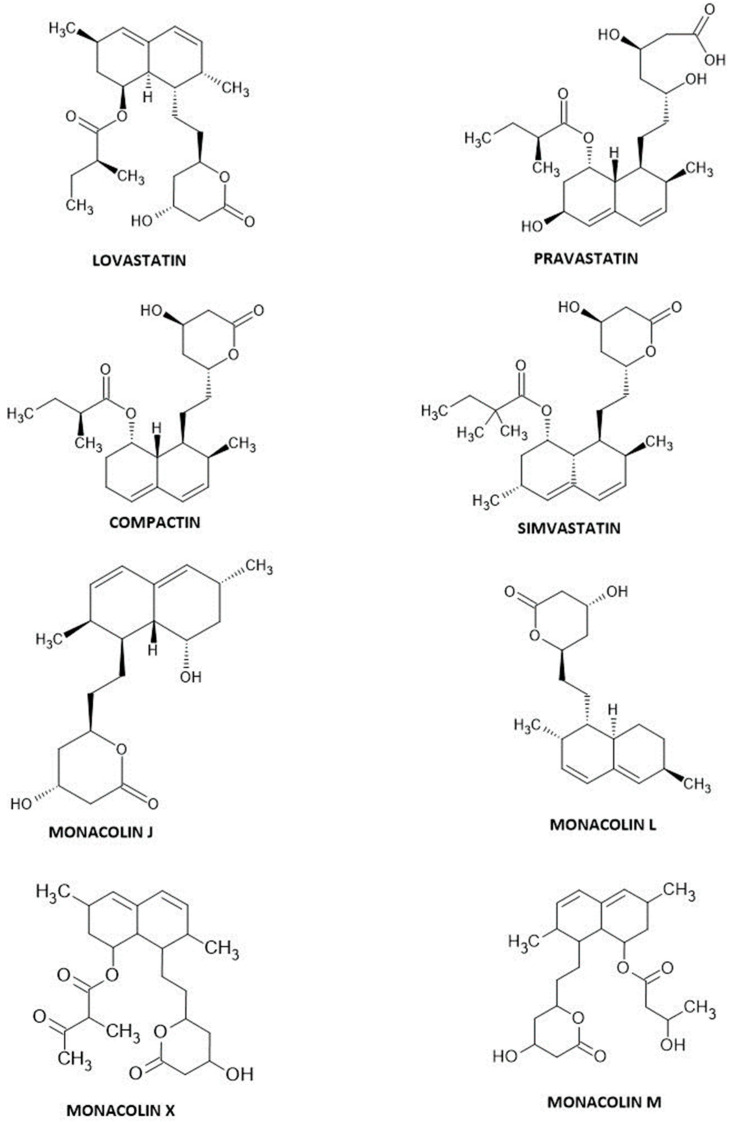
Statin structures.

Interestingly, in 1985, more monacolins were isolated and reported to have hypocholesterolemic effects. Firstly, monacolin J and monacolin L were isolated, then dihydromonacolin L, monacolin X, and monacolin M were unveiled from *Monascus ruber*, and monacolin L was also from *Aspergillus terreus*. It was alleged that these inhibitors were the secondary metabolites of biosynthesis in the process of fermentation, and they were structurally connected. Researchers showed that monacolin L was converted to monacolin J and, in turn, to monacolin K. It was observed that monacolin J was also converted to monacolin X, which was then transformed into monacolin K. Whereas monacolin M was derived synthetically from monacolin J differently than monacolins X and K [12,13,14]. The chemical structures of monacolins and fungal-derived statins are presented in Figure 1.

Plants are another source of monacolins. One example is red yeast rice (RYR), which is produced by fermenting rice with the red yeast Monascus purpureus and has the ability to lower blood lipid levels in both animals and humans [15,16]. RYR is recognized as a functional food with proven effectiveness in regulating hypercholesterolemia [12]. Monacolin K (MK) is the primary active component in RYR, as stated in studies by Lin et al. [17] and Perez-Jimenez et al. [18]. Researchers have conducted extensive investigations on MK to explore its potential physiological properties, such as neuroprotection [19], antibacterial and anti-inflammatory effects [20], and its possible use in anticancer treatments [21,22,23]. Despite their structural similarity, monacolin K and lovastatin exhibit different pharmacokinetic profiles and bioavailability. This may be attributed in part to the fact that lovastatin is administered as a singular active ingredient and thus has an oral bioavailability of approximately 30%, whereas monacolin K is just one component of fermented red rice, and other ingredients may modulate its bioavailability [24]. Studies have confirmed the effectiveness and tolerability of red yeast rice (RYR) in patients who cannot tolerate conventional statins. In a study by Stefanutti et al. [25], 55 patients with familial hypercholesterolemia who discontinued statins because of muscle pain were placed on a cholesterol-lowering diet including 300 mg of RYR (containing 10 mg of monacolin K) daily. After six months of treatment, the LDL cholesterol levels of both male and female patients decreased significantly (17% for men, 16% for women; *p* < 0.005). After 12 months, the levels decreased by 24% and 27% in men and women, respectively. None of the patients demonstrated elevated serum levels of aminotransferase or C-reactive protein [25].

The Food and Drug Administration (FDA) approved lovastatin in 1987, and this was the beginning of new statins on the market [26,27]. Pravastatin obtained FDA clearance in October 1991 [8,26], while simvastatin was cleared in December 1991 [26,27]. In 1993, the FDA gave the go-ahead for fluvastatin, which became the first synthetic statin [27]. At present, the most popular statin is atorvastatin, which was accepted by the FDA in December 1996 [1,27,28]. In 1997, cerivastatin was introduced to the market; however, it was subsequently withdrawn in August 2001 due to the high risk of rhabdomyolysis after its use [27,29,30]. Pitavastatin was launched on the Japanese market in September 2003, and the drug has been available in the United States since August 2009 [31]. Rosuvastatin has been on the market since 2003 [27]. 

## 3. Fungal-Derived Statins

**Figure 2:**
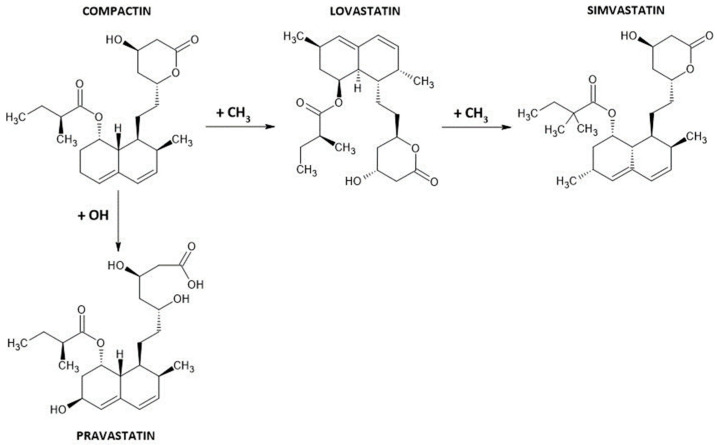
Differences in the chemical structures of fungal-derived statins.

Statins can be fungal-derived, semi-synthetic, or synthetic. The first group is comprised of drugs that were originally discovered in fungi, i.e., lovastatin and compactin. Their derivatives are pravastatin and simvastatin (Figure 2), while atorvastatin, cerivastatin, fluvastatin, pitavastatin, and rosuvastatin belong to fully synthetic compounds.

### 3.1. Lovastatin

A few fungal species are used for lovastatin production, and these are, for example, *Monascus* spp., *Penicillium citrinium*, *Pleurotus ostreatus*, or *Paecilomyces viridis*. Genomic and transcriptomic studies have generally been carried out on *A. terreus* ATCC 20542; therefore, *Aspergillus terreus* was the first to produce lovastatin [32].

Two types of lovastatin biosynthesis can be distinguished. The first one is SmF—liquid submerged fermentation; which is the cultivation of fungi in a nutrient medium with an excess of free-flowing water; and the second is SSF—solid-state fermentation; which is the cultivation process where fungi grow on solid materials without the presence of free liquid. SmF does not give as high results as SSF, which allows the production of 30 times more lovastatin from wild *Aspergillus terreus* than SmF under the same conditions. This is due to the correlation with a higher transcript of the genes LovE and LovF; the accumulation of the LovE transcript is 4.6-fold higher and the LovF transcript is 2-fold higher. It showed that the production of lovastatin in SSF depends on biosynthetic genes and higher levels of the LovE transcription factor. This correlates with higher production of other secondary metabolites in SSF [33,34]. Based on studies of environmental stimuli in the SSF, physiological changes are associated with increased lovastatin production, which made it possible to generate a mutant (OxB9) of *A. terreus* TUB F-514. As a result, 27.9 mg of lovastatin/g dc was produced using a high-density polyurethane foam (PUF) SSF system [8].

**Figure 3:**
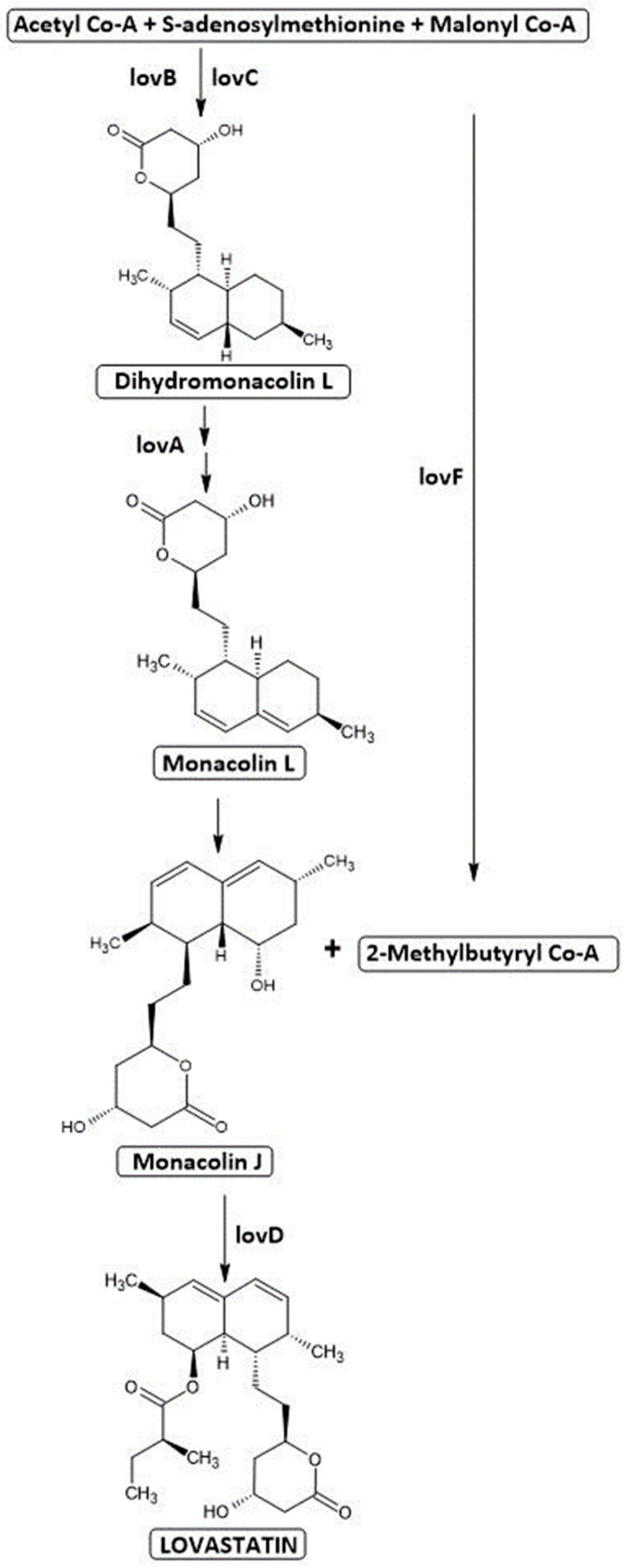
Lovastatin pathway. lovA—cytochrome P450; lovB (LNKS)—lovastatin nonaketide synthase; lovC—enoyl reductase; lovD—transferase; lovF (LDKS)—diketide synthase.

Lovastatin was obtained from a strain found in the soil in a laboratory in Madrid, Spain, and subsequently classified and deposited in the ATCC as *Aspergillus terreus* 20542 [7]. Lovastatin biosynthesis is based on the polyketide pathway. The LovB enzyme (lovastatin nonaketide synthase) internalizes 9 acetate molecules, while LovF (lovastatin diketide synthase) incorporates 2 molecules of acetate into a polyketide, and the merging of these two polyketides forms a lovastatin molecule (Figure 3). One acetyl-CoA and eight malonyl-CoA molecules undergo several condensation reactions to form a monacolin L. They assemble in a head-tail configuration, and the reaction is controlled by the LovB gene product [35]. Approximately 35 reactions are required to synthesize dihydromonacolin L. They are catalyzed by an enzyme encoded by the gene LovB together with the enoyl reductase enzyme (encoded by LovC) [32]. The next step is based on a cytochrome P450 monooxygenase (LovA), which catalyzes the conversion of dihydromonacolin L to monacolin L and finally to monacolin J. Meanwhile, the polyketide synthase LovF is involved in the synthesis of 2-methylbutyryl-CoA from acetyl-CoA and malonyl-CoA. In the final step, 2-methylbutyryl-CoA connects to monacolin J by a transferase encoded by LovD. The outcome of this reaction is the yield of lovastatin or mevinolinic acid [35]. Approximately 18 genes organized in 64 kb clusters are distinguished as being involved in lovastatin biosynthetic pathways. The most significant are the genes encoding lovastatin diketide synthase (LDKS) and lovastatin nonaketide synthase (LNKS). LDKS is composed of seven catalytic domains: KS, MAT, MT, ER, DH, ACP, and KR. It has been shown that the genes LovF, LovC, LovD, and LovA are substantial for the biosynthesis of lovastatin [32]. Other important genes include LovE and LovH, transcription factors encoding DNA-binding “zinc finger” domains [35,36].

It has been reported that the production of lovastatin is regulated by various physical and chemical factors, e.g., UV radiation, pH value regulation, glucose and lactose concentration, and nitrogen source.

One study demonstrated that exposure of *A. terreus* to UV radiation diminishes the production of secondary metabolites. This fact has been used to optimize lovastatin production. By using UV to perform random mutagenesis, a higher concentration of lovastatin was obtained. During the process, *A. terreus* NRRL265 has undergone mutagenesis via UV exposure, along with nitrous acid incubation to generate isolates. Among these isolates, one demonstrated a significantly higher concentration of lovastatin compared to NRRL265. As a result, this particular isolate was selected for media optimization. Eventually, eight times more lovastatin products were obtained due to strain and process modification [32].

It was also evidenced that efficient conditions occur with an alkaline or neutral pH of the medium, while the productivity of lovastatin was observed to decrease at lower pH values [37]. Consequently, increasing pH was shown to favor lovastatin production [38]. Additionally, a low level of glucose (20 or 45 g L^−1^) in the medium was found to favor a higher concentration of lovastatin. A study performed by Hajjaj et al. [39] showed that the addition of glucose to the culture of *A. terreus* significantly reduced the concentration of lovastatin; its production decreased when the glucose initial concentration was 70 g L^−1^. Moreover, *A. terreus* produced lovastatin at high residual lactose concentrations (approximately 25 g L^−1^) when grown on lactose, indicating that lovastatin synthesis is controlled by the cessation of the carbon source in addition to glucose suppression [39]. Bizukojc and Pecyna [40] studied the production of lovastatin in batch systems using lactose, glycerol, and a mixture of both. The results showed that lactose was more effective than glycerol, while the mixture had the highest concentration of lovastatin. Nonetheless, this type of production takes longer because lactose is absorbed after glycerol exhaustion. Additionally, nitrogen has been shown to impact the biosynthesis of lovastatin. Hajjaj et al. [39] showed that the addition of nitrogen sources like ammonium acetate, ammonium tartrate, ammonium nitrate, sodium nitrate, and urea minimized lovastatin production. Lai et al. [41] made a similar observation, which showed that ammonium sulfate applied as a nitrogen strongly decreased lovastatin production [41]. However, amino acids can also serve as a carbon and nitrogen source in filamentous fungi. Among amino acids, the best lovastatin production was acquired with cultures grown on sodium glutamate (12.5 g L^−1^) or histidine (12.5 g L^−1^); nonetheless, glutamate was chosen as the nitrogen source in the medium to allow rapid biomass formation due to its faster consumption [39]. On the other hand, Osman et al. [37] showed that methionine was the best amino acid to support the growth and productivity of lovastatin due to its direct involvement in the lovastatin biosynthetic pathway.

### 3.2. Simvastatin

Lovastatin enables the production of its half-synthetic derivative, simvastatin [42]. The synthesis of simvastatin starting from lovastatin is a multistep process, and various semisynthetic syntheses have already been described. The first method developed for commercial production of simvastatin entailed complete hydrolysis of lovastatin to achieve trihydroxy acid. The hydrolysis product was then heated to induce relactonization and to obtain dihydroxylactone as a result. The free hydroxy group, which is located in the lactone ring of the dihydroxylactone structure, is protected as a tert-butyldimethylsilyl ether. Then, 2,2-dimethylbutyryl chloride is applied to esterify the hydroxy group at the C-8 position of the hexahydronaphthalene ring system. The final step in simvastatin production involves removing the t-butyldimethylsilyl protecting group using tetrabutylammonium fluoride [43,44].

Another method of simvastatin synthesis involves the formation of an intermediate, monacolin J, during the hydrolysis of lovastatin. The acid is then lactonized to protect the C11 hydroxyl group and trimethylsilylated to give protection to the C13 hydroxyl. Monacolin J, which has undergone these procedures, is then acylated with α-dimethylbutyryl chloride, resulting in a protected form of simvastatin, which then undergoes chemical deprotection steps [45].

### 3.3. Pravastatin

The biosynthesis of pravastatin begins with the synthesis of compactin, a precursor molecule that undergoes enzymatic transformations to produce pravastatin. In order to enhance the production process of pravastatin, several research studies were conducted to increase the conversion rate of pravastatin by introducing high concentrations of compactin to the culture medium [46].

Compactin, also known as Mevastatin or ML-236B [47], is commercially produced by the fermentation of *Penicillium citrinum*, *Penicillium cyclopium*, and *Aspergillus terreus* [48]. The biosynthesis of compactin relies on a complex gene cluster from *P. citrinum*. It consists of nine genes, namely mlcA to mlcH, along with mlcR, which acts as the regulator, activating the expression of these genes. Researchers have identified the MlcR-binding sequence, which is found in the promoters of mlcA, mlcC, and other genes within the gene cluster. The introduction of additional copies of the regulatory gene mlcR has been observed to increase compactin production [8].

The structure of compactin consists of two distinct components: the hexahydro-naphthalene unit, forming its lower portion, and the lactone unit, forming the upper portion. The molecule exists in two forms: lactone and acid, with the acidic form being responsible for its biological activity [49,50]. Its isolation process is complex and involves lactonization of compactin, followed by multiple stages of solvent extraction and chromatography [8].

**Figure 4:**
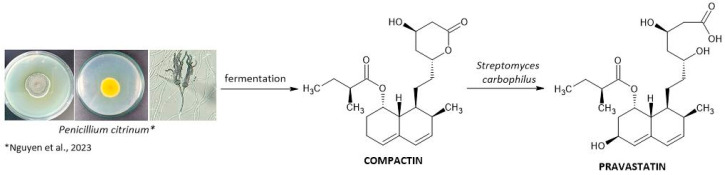
Pravastatin pathway [52].

Pravastatin synthesis starts with the condensation of acetyl-CoA and malonyl-CoA to produce compactin, which undergoes subsequent hydroxylation by various microorganisms, including *Nocardia*, *Amycolata*, *Saccharopolyspora*, *Amycolatopsis*, *Saccharothrix*, *Gilbertela*, *Actinomadura*, *Mortierella Nocardia*, and *Bacillus* spp. Several species of Streptomyces, such as *S. carbophilus*, *S. hastedi*, *S. flavovirens*, *S. rosenchromogenous*, *S. californicus*, and *S. exfoliatus*, are also known to carry out this bioconversion. The industrial two-step production process starts with the fermentation of *P. citrinum* to produce compactin (Figure 4). Secondly, the statins are purified, and the addition of sodium hydroxide opens the lactone ring. After neutralization, the open form of compactin undergoes conversion to pravastatin with the use of *Streptomyces carbophilus*. These bacteria contain a cytochrome P450 enzyme capable of stereospecific hydroxylation of compactin at C-6 [51].

The P450 enzyme is induced by the presence of compactin in the medium in concentrations preferably between 3 g/L and 6 g/L; hence, for efficient bioconversion, compactin must be added to the seed medium quite often (WO 98/45410). *Streptomyces flavidovirens* is a strain known to be highly efficient in the bioconversion of pravastatin, and according to Gururaja et al. [53], the optimal conditions for this process involve the following:

- A spore suspension or vegetative mycelium as the inoculum for the seed culture.
- Of 5% malt extract and peptone in the seed medium.
- pH of the seed medium between 6.0 and 7.5 before sterilization.
- Incubation of the seed medium at temperatures ranging from 25 to 35 °C for about 40 to 55 h.
- Selection of components such as dextrose monohydrate, peptone, and yeast extract for the production medium.
- Incubation of the production medium at temperatures between 24 and 35 °C for approximately 48 to 148 h.
- Using compactin, compactin salt, or compactin derivative as the substrate for bioconversion.
- pH regulation by feeding with a carbon source chosen from saccharides or glycerol [53].

In summary, achieving optimal conditions for pravastatin production requires considering the appropriate strain and inoculum, using specific compositions for the seed and production medium, adjusting pH levels, controlling incubation temperatures and durations, and utilizing suitable substrates and carbon sources for fermentation.

Pravastatin has gained significant attention due to its remarkable cholesterol-lowering effects and the potential to prevent cardiovascular events [8]. Among all the statins produced by microorganisms, it has significant advantages due to its stronger and highly tissue-selective inhibition of cholesterol synthesis [53,54].

## 4. Synthetic Statins

Over the past decades, numerous structural modifications of fungal-derived statins have been conducted to attain structurally improved and stronger derivatives. The researchers have focused on improving the efficacy of the pharmacokinetic profile for these drugs. The adjustments resulted in the development of structural modifications to the statin skeleton, and natural derivatives have been replaced with fully synthetic statins, which are often called super-statins. The structures of synthetic statins are disparate and also rather distinct chemically from those of natural statins. In fact, only the HMG-CoA-like moiety, which is responsible for the inhibition of HMG-CoA reductase, is mutually exclusive to all statins [55]. The chiral decalin core of natural analogs has been replaced by substituted heteroaromatic motifs in super-statins, whereas the β-hydroxy lactone moiety remained unchanged as it is crucial for their biological activity. Regarding structure, synthetic statins consist of a heterocyclic core linked to the chiral 3,5-dihydroxy-6-heptenoic or heptanoic acid side chain.

Super-statins represent fluvastatin, the first fully synthetic statin, followed by the most important synthetic statins—atorvastatin; rosuvastatin; and pitavastatin [56,57,58,59]. Cerivastatin, brought to the market in the 1990s, was withdrawn worldwide in 2001 due to the observed side effects [8].

## 5. Mechanism of Action

The mechanism of action of all statins is based on the interference with the conversion of HMG-CoA to mevalonate by HMG-CoA reductase, which is an early and rate-limiting step in the synthesis of cholesterol. Statins competitively and reversibly inhibit HMG-CoA reductase by connecting to this enzyme and inhibiting substrate binding due to the similarities of the statin pharmacophore to the moiety of HMG-CoA. Natural statins share a hexahydro-naphthalene moiety as the core structure, while synthetic ones have a completely different ring (a pyrrole-atorvastatin, an indole—fluvastatin; a pyrimidine—rosuvastatin; a pyridine—cerivastatin; and a quinoline—pitavastatin), and only the 3,5-dihydroxy-heptanoic acid segment is preserved, being the competitive analog of HMG-CoA [60]. On the other hand, the inhibiting effect of statin compounds depends on the strength of their bond with an enzyme. Moreover, the binding is also determined by the compound structure; e.g., the base structure of synthetic statins (i.e., cerivastatin, fluvastatin, atorvastatin, and rosuvastatin) containing fluorinated phenol groups provides additional sites for enzyme binding. Nevertheless, the differences in the structure are thought to impact statins’ bioactivity, determining their medical values, while the mechanistic relationship between medicinal properties and core structure has not been elucidated [61]. Evaluation of the crystal structures of the statin-enzyme complex showed that statins bind with HMGR with a large number of van der Waals forces. Moreover, it has been shown that rosuvastatin is distinguished by the largest quantity of binding interactions with the active site of HMG-CoA; similarly, rosuvastatin and atorvastatin stand out with an additional interaction with the enzyme, which was not seen in the case of other synthetic statins. It may explain the greater efficacy of these drugs in lowering LDL-C [62].

The inhibition of HMGR results in a reduction in cholesterol production. Ultimately, the decrease in cholesterol concentration leads to an up-regulation of LDL receptor expression, which promotes the consequent hepatic uptake of LDL from the bloodstream. Finally, the decrease of total cholesterol, triglycerides, and LDL and an increase of HDL characterize a cohesive lipid profile of all statins [63].

## 6. Pleiotropic Activity of Statins

**Figure 5:**
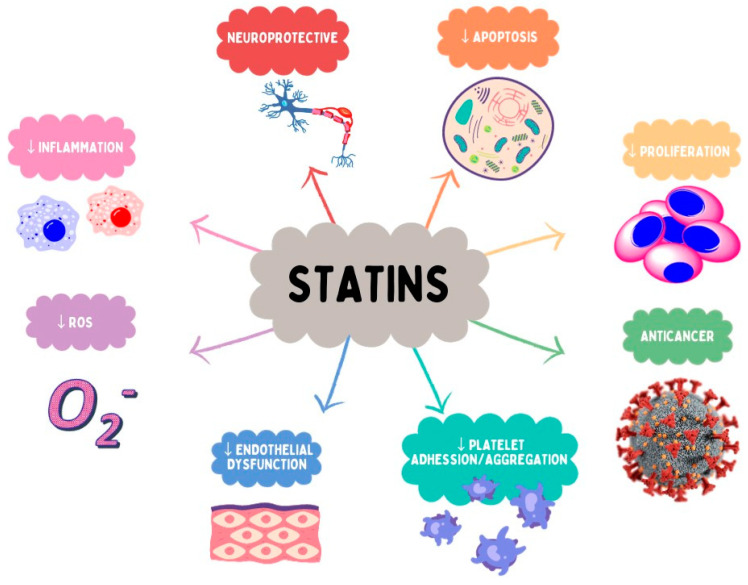
Selected pleiotropic effects of statins.

All statins are considered to effectively reduce LDL-C and triglyceride levels, as well as slightly increase HDL-C levels. The extent of the LDL-C lowering effect differs depending on the statin and its dose [64] (Table 1), through reversible and competitive inhibition of HMGR, which is involved in the biosynthesis of cholesterol and other sterols. Natural statins have low to moderate efficacy in reducing LDL-C in dyslipidemic patients, while atorvastatin and rosuvastatin, among the synthetic drugs, have the highest intensity according to the AHA/ACC Classification of Intensity [65]. Although the principal mechanism of statins is possibly engaged in the prevention of stroke and coronary events, there are other benefits partly related to other mechanisms. These favorable effects represent, among others, an antiproliferative influence on smooth muscle cells, reconstruction of endothelial activity, antioxidant, antithrombotic, anticancer, and anti-inflammatory effects. The above-mentioned features are referred to as pleiotropic effects (Figure 5) [66,67,68]. Statins have been demonstrated to reduce the risk of cardiovascular morbidity and mortality; they also possess the ability to prevent stroke as well as reduce the development of peripheral vascular disease [66]. Over the years, there has been evidence suggesting that statins might provide benefits in several diseases of the central nervous system, particularly Alzheimer disease (AD) or vascular dementia [69,70,71].

In the era of the coronavirus (COVID-19) pandemic, numerous studies have been conducted to develop effective strategies for managing the effects of viral infection and reducing its morbidity and mortality. Infection with SARS-CoV-2 can also lead to cardiovascular complications [103], such as stroke, arrhythmias, venous thromboembolism, myocardial injury, myocarditis, pericarditis, cardiomyopathy, or cardiogenic shock. The main receptor responsible for SARS-CoV-2 transmission is angiotensin-converting enzyme 2 (ACE2), a regulatory enzyme of the renin-angiotensin system. The virus binding to ACE2 on the surface of lung cells mediates entry and infection consequently [51,104,105,106]. It is believed that statins play an important role in the treatment of COVID-19. Daniels et al. [107] showed that patients taking statins before hospitalization for COVID-19 demonstrated a 16% lower risk of death in comparison to those who did not use statins. Some meta-analyses have reported beneficial outcomes for statin users, such as a reduced risk of fatal or severe disease [108] and a reduced likelihood of progression to severe disease or death [109]. Further randomized-controlled trials will be helpful in the elaboration of statins’ role in SARS-CoV-2 infection [110]. Selected clinical trials regarding the beneficial and pleiotropic effects of statins are presented in Table 2.

## 7. Selected Aspects of Statin Pharmacokinetics

Simvastatin and lovastatin are administered as prodrugs in the form of inactive lactones, which are transformed into active forms in the liver. Other statins, such as fluvastatin, pravastatin, atorvastatin, rosuvastatin, and pitavastatin, are applied in the active acid form [150]. Even though atorvastatin and cerivastatin are characterized by pharmacologically active parent drugs, they may also be biotransformed to yield metabolites contributing to their lipid-lowering effects [66].

Differences in lipophilicity or hydrophilicity among statins may affect drug kinetics and tissue selectivity. Pravastatin and rosuvastatin, in comparison to other statins, exhibit relatively low lipophilicity, and being more hydrophilic, they remain associated with the polar membrane surface. Consequently, protein transporters are necessary to enter the cell and inhibit the HMG-CoA reductase enzyme [151]. The lipophilic statins, i.e., simvastatin, pitavastatin, fluvastatin, lovastatin, and atorvastatin, can easily pass into the membranes and interact with the surrounding acyl chains [62,152,153].

Almost all statins bind highly (around 90%) to serum proteins, especially albumin [153], with the exception of pravastatin, which is only about 50% bound. Consequently, pravastatin is less likely than other statins to dissociate from albumin-drug binding.

The lipophilic statin lactones are metabolized more quickly by the cytochrome P450 enzymes than are the statin acids [154]. Carboxylesterases are responsible for the conversion of lactones in the liver, the gut wall, and partly in plasma, and the response is completely reversible. The open acid form of statins, regardless of whether it is administered as a lactone or an acid, can be converted to the lactone form by glucuronidation [155]. Lactone formation may be mediated indirectly by uridine diphosphate glucuronosyltransferase (UGT) enzymes [154]. UGT conjugates with open acids to give an acyl glucuronide that is cyclized to form a lactone ring. As an effect, the loss of pharmacological activity affects all statins in the open acid form [156].

Statins are metabolized in the liver by CYP 450 isoenzymes, except for pravastatin, which undergoes sulfation in the cytosol of liver cells [157,158]. CYP3A4 is a major contributor to the metabolism of lovastatin, simvastatin, and, to a lesser extent, atorvastatin. CYP3A4 and CYP2C8 are responsible for the metabolism of cerivastatin [62]. Rosuvastatin and pitavastatin are only slightly metabolized by the isoenzyme CYP450 and therefore exhibit a lower risk of interaction. The principal metabolism pathway of pitavastatin involves lactonization/glucuronidation [157]. Less than 10% of rosuvastatin is metabolized by the CYP2C9 enzyme, while fluvastatin is predominantly metabolized by CYP2C9 and, to a lesser extent, by CYP2C8 and CYP3A4 [158]. Because of rapid metabolism in the gut and liver, the statins’ bioavailability is generally low, except for cerivastatin and pitavastatin (Table 3).

It is known that statins are substrates for organic anion-transporting polypeptides (OATP). The transport of simvastatin, atorvastatin, pravastatin, pitavastatin, fluvastatin, and rosuvastatin mediated by OATP1B1 showed apparent Km values ranging from 0.6 to 29 μM, where atorvastatin demonstrated the highest and pravastatin the lowest affinity for OATP1B1 [155]. Some drugs, such as cyclosporine, inhibit OATP and may, therefore, increase the plasma concentration of atorvastatin and some other statins [159]. Although pravastatin is not metabolized by the isoenzymes of cytochrome P450, there is evidence that it interacts with macrolides such as erythromycin and clarithromycin. This interaction may involve uptake transporters. Macrolides are inhibitors of the uptake of drugs, mediated by OATPs, which serve as an additional mechanism of interaction [160]. The risk of myopathy may be increased by cyclosporine protease inhibitors, which also inhibit OATP1B1 [161].

The intestinal and biliary elimination of certain statins is mediated by P-glycoprotein (P-gp), an efflux transporter localized in a variety of tissues, including the gastrointestinal tract, liver, and kidneys. Only lovastatin, simvastatin, pitavastatin, and atorvastatin are substrates of p-glycoprotein (P-gp) [150,157].

**Table 3:** Statin pharmacokinetic properties comparison [62,66,150,157,159,162,163,164].

Drug	Prodrug	Solubility	Bioavailability (%)	Metabolism by Cytochrome P450 Enzymes	OATP Transport	Substrates of P-glycoprotein	Metabolites	Protein Binding	T1/2 (h)
**Type I—fungal derived statins**									
lovastatin	Yes	Lipophilic	<5	CYP3A4	OATP1B1	Yes	Active metabolites	>96%	2–4
pravastatin	No	Hydrophilic	17	Sulfation	OATP1B1 OATP1B3 OATP2B1	No	Not active metabolites	50%	1–3
simvastatin	Yes	Lipophilic	<5	CYP3A4	OATP1B1	Yes	Active metabolites	>95%	2–3
**Type II—synthetically derived statins**									
fluvastatin	No	Lipophilic	24	CYP2C9	OATP1B1 OATP1B3 OATP2B1	No	Not active metabolites	>98%	0.5–3
atorvastatin	No	Lipophilic	~12	CYP3A4	OATP1B1 OATP2B1	Yes	Active metabolites	>98%	15–30
cerivastatin	No	Lipophilic	60	CYP3A4 CYP2C8	OATP1B1	No	Active metabolites	>99%	2–3
pitavastatin	No	Lipophilic	~60	Main: lactonization/glucuronidation Minimal: CYP2C8 CYP2C9	OATP1B1 OATP1A2 OATP1B3	Yes	Minimally metabolized	96%	10–12
rosuvastatin	No	Hydrophilic	20	Minimal: CYP2C9 CYP2C19	OATP1B1 OATP1A2 OATP1B3 OATP2B1	No	Minimally metabolized	88%	19–20

CYP—cytochrome P450 enzyme; OATP—Organic anion transporting polypeptide.

## 8. Interactions

The use of statins is increasing and currently affects about a quarter of the world’s population. Individuals aged 65 and above take statins on a long-term basis to prevent primary and secondary cardiovascular disease (CVD) [165]. Consequently, patients taking multiple medications and at risk of drug-drug interactions are particularly concerned about the safety and side effects of statins. Many significant drug interactions are pharmacokinetic in nature, and their level of risk varies among various statins. This may affect the safety and tolerance of statins to different extents [62,166].

As cytochrome CYP3A4 is commonly involved in the metabolism of many drugs, statins, which are primarily metabolized by this enzyme, are susceptible to drug interactions. Drugs that inhibit CYP3A4 may increase statin concentrations in the bloodstream, elevating the risk of drug toxicity. Meanwhile, substrates of the enzyme system can also heighten systemic statin concentrations due to competition with statins within the same metabolic pathway. Selected inhibitors and inducers of CYP3A4 and CYP2C9 are presented in Table 4 [162,167].

Finally, not only prescribed medications but also other over-the-counter drugs, as well as vitamins, minerals, and herbal foodstuffs, contribute to increased interaction risk. Optimal CYP450 activity is an important determinant of statin metabolism, while some substances, such as grapefruit juice, interfere with the action of CYP3A4 [162,167]. In a study to investigate the effect of grapefruit juice on the pharmacokinetics of simvastatin, 10 healthy people were given 200 mL of double-strength grapefruit juice or water three times a day over two days. On the next day, each patient took a dose of 60 mg of simvastatin in combination with 200 mL of grapefruit juice or water, and another 200 mL of juice was taken half an hour and one and a half hours after the statin was administered. Under the influence of the juice, the levels of serum simvastatin and simvastatin acid rose. This study showed that taking simvastatin and grapefruit juice should be avoided at the same time [169]. Ethanol also undergoes metabolism by cytochrome P450. Hence, the ingestion of statins while drinking alcohol nearby may lead to hepatic damage [170].

## 9. Adverse Reactions

Statins are generally well-tolerated, and the most common serious adverse reactions include muscle-related symptoms, hepatotoxicity, renal toxicity, and type 2 diabetes mellitus. Other side effects include skin rashes, muscle abnormalities, and gastrointestinal symptoms. The mechanism of statin toxicity is thought to arise due to HMGR inhibition, direct cellular and subcellular effects, or a combination of both, as well as genetic factors, drug interactions, vitamin D deficiency, or immune disorders. Moreover, adverse side effects are class-, dose-, time-, age-, or sex-dependent.

The most prevalent and significant adverse reactions are muscle-related, manifested as myalgia, myopathy, myositis with elevated creatine kinase, or the most severe, rhabdomyolysis [171]. These are typically the primary reasons for the statin discontinuation. Pain, muscle weakness, increased creatine kinase levels, and rhabdomyolysis have been reported [172]. Studies on the muscle toxicity of statins were carried out on mice, rabbits, and rats [173,174]. Research has documented the harmful effects of combining gemfibrozil with statins, as gemfibrozil increases the concentration of statins in plasma. It also raised the peak concentration (Cmax) and extended the half-life (T1/2) of simvastatin, pravastatin, and cerivastatin. These interactions may lead to myopathy when using both drug groups simultaneously [175]. Changes in the stability and fluidity of muscle cell membranes may be responsible for these symptoms. Furthermore, this interaction could have a negative impact on protein signaling and activity, as well as mitochondrial function [176].

Statin treatment may potentially cause liver damage, as evidenced by a significant increase in alanine and aspartate aminotransferases to three times above normal levels. Symptoms can emerge during the first three months of treatment. It is recommended to closely monitor liver function in patients undergoing statin treatment. The examination should be repeated twice, and the therapy should be discontinued if it is necessary, allowing aminotransferase activity levels to fall within 2–3 months [177]. The increase in their concentrations is not a result of histopathological changes or toxic liver damage, as with elevated bilirubin. In this case, statin therapy should be discontinued immediately and liver function monitored. Patients with chronic liver failure and chronic hepatitis non-alcoholic steatosis do not have an increased risk of liver damage [178]. During animal studies, it has been observed that the reduction of mevalonate or its intermediates can increase liver enzyme activity. In addition, one hypothesis to explain their increased concentration without histopathologic changes is the altered lipid composition of the hepatic cell membrane. This leads to a change in their permeability and enzyme release [179].

The brain is an organ that accounts for approximately 20% of the total cholesterol in the human body. Accurate control of cholesterol homeostasis in the brain is necessary for proper function, and imbalances in brain cholesterol homeostasis can have significant consequences [180,181,182,183]. Recently, neurologic adverse events associated with statin therapy were raised [184,185,186]. Published studies based on individual case reports have provided different and conflicting conclusions [187]. Clinical trials have investigated the therapeutic potential of statins for treating central nervous system disorders, including dementia, multiple sclerosis (MS), depression, stroke, and epilepsy. Retrospective studies and meta-analyses have examined the incidence of various neurological conditions after statin treatment [188,189,190]. In addition, the FDA has emphasized the need for new data on the potential psychiatric effects of all statins, and ongoing studies have focused on recording the neurocognitive side effects induced by statins according to solubility profiles [152,191,192]. Lipophilic statins directly affect brain cholesterol metabolism by crossing the blood-brain barrier and inhibiting neuronal cholesterol synthesis [193].

Lowering cholesterol levels in the brain cell membrane may be a contributing factor to the side effects of statins, such as aggression, depression, emotional lability, nervousness, panic, amnesia, insomnia, and hallucinations. Simvastatin accounted for 21% of all reported side effects, with the majority occurring in females. It has been observed that other lipid-lowering drugs have similar side effects to those of statins [194]. Pop et al. [195] reported that the number of adverse reactions related to the System Organ Class (SOC) ‘Psychiatric Disorders’ were higher for atorvastatin, simvastatin, and rosuvastatin compared to other statins, according to data submitted to the EudraVigilance database [195].

Olson et al. [196] investigated the relationship between lipid-lowering agents and aggressive behavior, hostility, cynicism, and depression scores in women undergoing coronary angiography. This study found that women taking statins had higher aggressive behavior scores than those not treated with lipid-lowering drugs. Statin use was an independent factor in predicting the aggression scores in regression analyses. Several studies have found a correlation between low serum cholesterol levels and depression [197,198,199], suicidal behavior [193,199,200,201,202], impulsivity, and aggression [203].

Statin-associated psychiatric effects are rare and inevitable events that are most likely to be seen in susceptible patients with subclinical impairment of neurotransmitter pathways. In the majority of cases, symptoms resolved spontaneously upon discontinuation of statins [194,204,205,206,207].

## 10. Summary

Statins, which are selective inhibitors of HMGR, are the primary drugs recommended for the pharmacological treatment of dyslipidemia and hypercholesterolemia. They are categorized based on their origin as natural, semisynthetic derivatives, or synthetic. Fungi are the source of statins. Natural statins are derived from the fermentation processes of fungi and molds, such as *Monascus* spp., *Penicillium* spp., *Aspergillus tereus*, and *Pleurotus ostreatus*. Fungal-derived statins are lovastatin, pravastatin, and simvastatin. The initial compound, known as mevastatin, was utilized as a precursor for the production of pravastatin via enzymatic conversion. It has been a key compound in the development of synthetic statins used today, such as fluvastatin, pitavastatin, rosuvastatin, and atorvastatin, through multi-step chemical processes. Synthetic statins differ in their structural composition from natural statins. The sole common feature between natural and synthetic statins is the HMG-CoA-like moiety, responsible for inhibiting HMGR. There is literature discussing the pleiotropic effects of statins, which extend beyond cholesterol reduction. Nevertheless, these medications exhibit differences in pharmacokinetic parameters, including the potency of enzyme blockade and duration of action. Fungi, which are the source of statins, are often studied for their extensive biological effects to discover novel therapeutic alternatives.

## Figures and Tables

**Table 1:** Effects of Different Types of Statin on Lipid Profile.

Drug	LDL-C	HDL-C	TG	Adverse Effects and Dosage Characteristics
**Type I—fungal derived statins**				
**lovastatin**	20 mg/day—Low intensity dosage 40–80 mg/day—Moderate intensity dosage [65] ↓ 17%—10 mg/day [72] ↓ <30%—20 mg/day [73] ↓ 24%—20 mg/day [72] ↓25%—20 mg/day [74] ↓ 25%—40 mg/day [75] ↓ 27%—40 mg/day [72] ↓ 30–50%—40–80 mg/day [73]	↑ 8.3%—20 mg/day [74]	↓10.9%—20 mg/day [74] ↓ 30%—40 mg [75]	It is advisable to take the medication in the evening with a meal. Safety and effectiveness in children have not been established [76].
**pravastatin**	10–20 mg/day—Low intensity dosage 40–80 mg/day—Moderate intensity dosage [65] ↓ 23%—5 mg/day and 10 mg/day [77] ↓ 33%—20 mg/day [77] ↓ 25.4%—40 mg/day [78]	Not observed significant changes—40 mg/day [78] Not observed significant changes—5 mg/day and 10 mg/day [77] ↑ 11%—20 mg/day [77]	Not observed significant changes—40 mg/day [78] Not observed significant changes—5 mg/day, 10 mg/day, and 20 mg/day [77]	Recommended intake in the evening. To reduce hyperlipidemia in organ transplant patients receiving immunosuppressive therapy [79].
**simvastatin**	10 mg/day—Low intensity dosage 20–40 mg/day—Moderate intensity dosage [65] ↓ 20–30%—10 mg/day [27] ↓ 30–40%—20 mg/day [27] ↓ 40–45%—40 mg/day [27] ↓ 46–50%—80 mg/day [27] ↓ 49%—80 mg/day [80]	↑ 4.2%—10 mg [81] ↑ 5.3%—80 mg [81] ↑ 6%—80 mg/day [80]	Falling to 35%—dose 80 mg/day [80]	Recommended intake in the evening [82]. If the activity of hepatic organic anion transport proteins is reduced, there may be an increased risk of myopathy and rhabdomyolysis. These result from an increase in simvastatin acid exposure. Those who take interacting drugs or who carry the SLCO1B1 c.521T > C genotype are most at risk [83].
**Type II—synthetically derived statins**				
**fluvastatin**	20–40 mg/day—Low intensity dosage 40 mg 2x/day; 80 mg XL—Moderate intensity dosage [65] ↓ 15% to 33%—10 mg/day to 80 mg/day [84] ↓ 22–36%—20–80 mg/day [85]	↑ 3.3–5.6%—20–80 mg/day [85]	↓ 3%–7.5%—10 mg/day–0 mg/day [84] ↓12–18%—20–80 mg/day [85]	In the case of 20 mg and 40 mg doses, it is recommended to take the tablet in the evening; in the case of 80 mg, any time of day [86].
**atorvastatin**	10–20 mg/day—Moderate intensity dosage 40–80 mg/day—High intensity dosage [65] ↓ 35%—10 mg/day [87] ↓ 30–40%—10 mg/day [27] ↓ 40–45%—20 mg/day [27] ↓ 46–50%—40 mg/day [27] ↓ 50–55%—80 mg/day [27] ↓ 37.1%—51.7%—10–80 mg/day [88] ↓ 36% to 53%—10–80 mg/day [89]	↑ 8%—10 mg/day [87] ↑ 4.5%—10 mg [81] Falling to 2.3%—80 mg [81]	↓ 20%—10 mg/day [90] ↓ 22.6%—20 mg/day [90] ↓ 26.8%—40 mg/day [90]	Taking a statin at any time of the day [91]. A dose of 80 mg increases the risk of hamorrhagic stroke after a stroke, or TIA [92].
**cerivastatin**	↓ 11%—40.8%;—0.025–0.8 mg/day [93] ↓ 37.3%—0.4 mg/day [94] ↓ 42.2%—0.8 mg/day [94]	↑ 5%— dose-independent [93]	↓ 9%—21.4%—0.025–0.8 mg/day [93]	Due to the high risk of rhabdomyolysis, it is presently not used [29].
**pitavastatin**	1–4 mg/day—Moderate intensity dosage [65] ↓ 33.3%–4.7%—1 mg/day–6 mg/day [95]	There was no evidence of a dose-dependent effect of pitavastatin on blood HDL. cholesterol levels. An average increase of 4% (for all doses) [95] ↑ 8.2%—2 mg/day [96] ↑ 20.1%—2–4 mg/day [96] ↑ 6.3%—4 mg/day [96]	↓ 13.0%–8.1%—1 mg/day–6 mg/day [95]	Taking a statin at any time of the day. Not for use under age 18 [97] It causes a higher increase in HDL-C than other statins. This level is maintained over a longer period of time. Its characteristic feature is its metabolism, which avoids interactions with other drugs (e.g., no interaction with warfarin) [31]. Due to its pleiotropic effects, it contributes to the stabilization of the coronary plaque, reduces the migration of monocytes, and promotes the formation of foam cells [98]. The only statin capable of plasma adiponectin elevation is [96].
**rosuvastatin**	5–10 mg/day—Moderate intensity dosage 20–40 mg/day—High intensity dosage [65] ↓ 40%—5 mg/day [87] ↓ 30–40%—5 mg/day [27] ↓ 43%—10 mg/day [87] ↓ 40–45%—5–10 mg/day [27] ↓ 46–50%—10–20 mg/day [27] ↓ 50–55%—20 mg/day [27] ↓ 53%—40 mg/day [99] ↓ 56–60%—40 mg/day [27] ↓ 46%—55%;—10–40 mg/day [100]	From 5.5% to 7.9%; dose 5–40 mg/day [81] ↑ 13%—5 mg/day [87] ↑ 12%—10 mg/day [87]	↓19.8%—10 mg/day [15] ↓23.7%—20 mg/day [15] ↓26.1%—40 mg/day [15]	Taking a statin at any time of the day, Statins can be used from the age of 6 [101]. It has the strongest hypolipemic effects; for example, a dose of rosuvastain of 5–10 mg has the same effect as 20–30 mg of atorvastain. There is no significant effect on the risk of renal failure in people without renal impairment [99]. In renal impairment in patients with creatinine clearance (CrCl) < 30 mL/min, it is recommended to start with the lowest possible dose of 5 mg/day with a max dosage of 10 mg/day; if the CrCl ≥ 30 mL/min, no dosage adjustment is necessary [102].

LDL-C—low-density lipoprotein cholesterol; HDL-C—high-density lipoprotein cholesterol; TG—triglycerides; LDL-C lowering: <30%—Low Intensity; 30–49%—Moderate Intensity; ≥50%—High Intensity, according to the 2023 AHA/ACC/ACCP/ASPC/NLA/PCNA Guideline for the Management of Patients With Chronic Coronary Disease: A Report of the American Heart Association/American College of Cardiology Joint Committee on Clinical Practice Guidelines. Circulation [65].

**Table 2:** Selected beneficial and pleiotropic effects of different types of statins based on clinical trials.

Drug	Clinical Effects/ the Clinical Trial Number	Pleiotropic Effects/ the Clinical Trial Number
**Type I—fungal derived statins**		
**lovastatin**	reduction of LDL cholesterol level/NCT03242499 [111], NCT04359823 [112]	the effect on synaptic plasticity, cognitive function, and attention in patients with RASopathies/NCT03504501 [113]
		improvement in the treatment of diffuse superficial actinic porokeratosis/NCT04359823 [112]
		modulation of cortical inhibition in neurofibromatosis type 1 (NF1)/NCT03826940 [114]
		reduction of motor symptoms in patients with early-stage Parkinson’s/NCT03242499 [111]
**pravastatin**	reduction of LDL cholesterol level/ACTRN12616000535471 [115]	prevention of pre-eclampsia in pregnant women/NCT01717586 [116]
		biomarker for monitoring the clinical benefit of statin treatment in secondary prevention/ ACTRN12616000535471 [115]
		prevention of the occurrence of atherothrombotic brain infarction in noncardioembolic infarction patients/NCT00361530 [117]
		reducing the incidence of lacunar stroke/NCT00221104 [118]
		reduction in levels of lipoprotein subclasses/NCT03073018 [119]
		reduction of vascular inflammation in non-cardiogenic ischemic stroke/NCT00361699 [120]
**simvastatin**	inhibition of 3-hydroxy-3-methyl-glutaryl- CoA reductase/ NCT00529139 [121] reduction in serum LDL concentration/NCT00939822 [122], NCT01061671 [123]	activation of PI3K-kinase/NCT00676897 [121]
		positive effect on emotions/NCT04652089 [124]
		improved survival in ever-smokers with extensive disease (ED)–small cell lung cancer (SCLC)/NCT01441349 [125]
		reductions in total cholesterol, lipoprotein, and triglycerides observed in children with type 1 diabetes mellitus/NCT03660293 [126]
		anti- inflammatory and neuroprotective effects/NCT01999309 [127]
		prevention of decompensation in patients with compensated cirrhosis/NCT03654053 [128]
**Type II—synthetically derived statins**		
**fluvastatin**	reduction in blood lipid levels/ ChiCTR-TRC-12002642 [129] decrease in LDL-level/NCT00421005 [130]	decrease of inflammatory index, ultrasonic index and electrocardiographic measurement results in atrial fibrillation/ChiCTR-TRC-12002642 [129]
		normalization of bilirubin levels and reduced blood lipids in patients with nephrotic syndrome/ChiCTR-TQR-12002602 [131]
		preventing pathologic changes in graft coronary arteries/NCT00421005 [130]
**atorvastatin**		reduction of levels of inflammatory markers, such as CD62-L-selectin, matrix metalloproteinases-2, and TNF-α/NCT04072601 [132]
		acceleration wound healing and alleviating pain from laparotomy surgical wounds/IRCT20190810044500N3 [133]
	reduction of the coronary plaque/NCT00965185 [134] reduction of LDL cholesterol level/NCT02579499 [135]	prevention of adverse cardiovascular outcomes, particularly carotid intimal thickening, in HIV-infected patients on viral suppression/NCT04101136 [136]
		reduction of the incidence of cardiac dysfunction among patients with lymphoma treated with anthracycline-based chemotherapy/NCT02943590 [137]
		prevention of contrast-induced nephropathy in patients with chronic kidney disease undergoing angiography/IRCT20190810044500N3 [138]
**pitavastatin**	reduction of LDL-C level/NCT04289649 [139], NCT00846118 [140], NCT04584736 [141]	lower risk of major adverse cardiovascular articipants with HIV infection/NCT02344290 [142]
		increase of PCOLCE (enzymatic cleavage of type I procollagen) and decrease of PLA2G7 (systemic marker of arterial inflammation) in patients with HIV/NCT01301066 [143], anti-inflammatory effects in people with HIV/NCT02442700 [144]
		Reduced mortality in dyslipidaemic patients on chronic haemodialysis/NCT00846118 [140]
		reduce insulin hepatotoxicity/NCT02290106 [145]
**rosuvastatin**	reduction of LDL-C level/NCT04826354 [146], NCT03044665 [147], NCT03951207 [148]	improved lipid profiles and reduction of vascular inflammation/NCT02749994 [149]

**Table 4:** Selected inhibitors and inducers of cytochromes CYP3A4 and CYP2C9 [162,166,167,168].

Enzyme	CYP2C9	CYP3A4
Statin substrates	Fluvastatin, rosuvastatin	Atorvastatin, lovastatin, and simvastatin
Inducers	Carbamazepine, phenobarbital, phenytoin, and rifampin	Aprepitant, carbamazepine, cyclophosphamide, corticosteroids, efavirenz, nevirapine, phenytoin, pioglitazone, phenobarbital, and St. John’s wort
Inhibitors	Amiodarone, capecitabine, fluconazole, fluvoxamine, ketoconazole, metronidazole, miconazole, sulfamethoxazole/trimethoprim, voriconazole, zafirlukast	Amiodarone, clarithromycin, cyclosporine A, diltiazem, erythromycin, fluconazole, fluoxetine, fluvoxamine, grapefruit juice, isoniazid, itraconazole, ketoconazole, mibefradil, midazolam, nefazodone, protease inhibitors, sertraline, tacrolimus, ticagrelor, tricyclic antidepressants, verapamil

## Data Availability

Not applicable.
